# 11C-methionine-PET for differentiating recurrent brain tumor from radiation necrosis: radiomics approach with random forest classifier

**DOI:** 10.1038/s41598-019-52279-2

**Published:** 2019-10-30

**Authors:** Masatoshi Hotta, Ryogo Minamimoto, Kenta Miwa

**Affiliations:** 1 Department of Radiology, National Center for Global Health and Medicine, 1-21-1, Toyama, Shinjuku-ku, Tokyo, 162-8655 Japan; 2 Department of Radiological Sciences, School of Health Science, International University of Health and Welfare, 2600-1 Kitakanemaru, Ohtawara city, Tochigi 324-850 Japan

**Keywords:** CNS cancer, Neurology

## Abstract

Differentiating recurrent brain tumor from radiation necrosis is often difficult. This study aims to investigate the efficacy of 11C-methionine (MET)-PET radiomics for distinguishing recurrent brain tumor from radiation necrosis, as compared with conventional tumor-to-normal cortex (T/N) ratio evaluation. We enrolled 41 patients with metastatic brain tumor or glioma treated using radiation therapy who underwent MET-PET. The area with a standardized uptake value > 1.3 times that of the normal brain cortex was contoured. Forty-two PET features were extracted and used in a random forest classifier and the diagnostic performance was evaluated using a 10-fold cross-validation scheme. Gini index was measured to identify relevant PET parameters for classification. The reference standard was surgical histopathological analysis or more than 6 months of follow-up with MRI. Forty-four lesions were used for the analysis. Thirty-three and 11 lesions were confirmed as recurrent brain tumor and radiation necrosis, respectively. Radiomics and T/N ratio evaluation showed sensitivities of 90.1% and 60.6%, and specificities of 93.9% and 72.7% with areas under the curve of 0.98 and 0.73, respectively. Gray level co-occurrence matrix dissimilarity was the most pertinent feature for diagnosis. MET-PET radiomics yielded excellent outcome for differentiating recurrent brain tumor from radiation necrosis, which outperformed T/N ratio evaluation.

## Introduction

Surgery, chemotherapy, and radiation therapy are essential for the treatment of malignant brain tumors, including glioma and brain metastasis. After conventional radiotherapy and stereotactic radiosurgery, radiation necrosis can occur^1^. Accurate diagnosis of radiation necrosis is crucial because of its great influence on subsequent management^2^. However, radiation necrosis often mimics recurrent brain tumor on gadolinium-enhanced MRI due to the breakdown of the blood-brain barrier under both pathological states, and differentiation of these two states is thus difficult^3^. Many reports have demonstrated the advantages of 11C-methionine (MET)-PET in differentiating radiation necrosis form recurrent brain tumor^4^. When assessing MET-PET, tumor-to-normal cortex uptake (T/N) ratio is commonly used, calculated as the maximum standardized uptake value (SUVmax) of the lesion divided by the mean SUV (SUVmean) of the normal frontal brain cortex^5–7^. However, T/N ratio reflects only signal voxel uptake (i.e., SUVmax) of the lesion and does not include volume-based information, and thus can still show false-positive or false-negative outcomes.

Recently, texture analysis as a volume-based analysis quantifying tumor properties beyond the capability of visual interpretation or simple metrics has been identified as an essential tool for “radiomics”^8,9^. Radiomics is a high-throughput approach for extracting a large number of quantitative features, including spatial tumoral heterogeneity, which is deeply associated with cellular and molecular characteristics such as cellular proliferation and necrosis^10,11^. The value of radiomics with 18F-fluorodeoxyglucose (FDG)-PET for differential diagnosis and prognosis prediction has been reported for several malignancies, such as lung, breast, esophageal, and cervical cancers, and soft-tissue tumors^12–17^. In addition, some recent radiomics studies have used machine-learning methods like support vector machines, neural networks, and random forest classifiers^18–20^, enabling more robust statistical analysis^8^. However, the efficacy of radiomics using MET-PET for malignant brain tumor has not been investigated.

The aim of this study was to evaluate the diagnostic value of MET-PET radiomics using a random forest classifier for differentiating between radiation necrosis and recurrent brain tumor, as compared with differentiation from T/N ratio evaluations.

## Results

**Figure 1:**
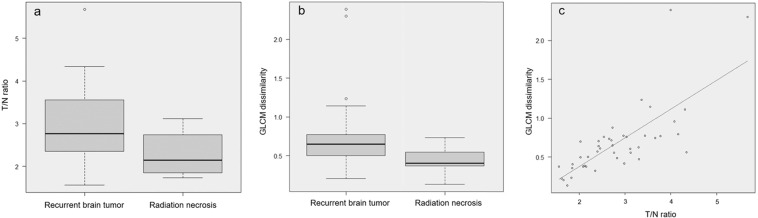
T/N ratio (**a**) and gray-level co-occurrence matrix (GLCM) dissimilarity (**b**) compared between recurrent brain tumor and radiation necrosis. (**c**) Spearman’s correlation coefficients for T/N ratio and GLCM dissimilarity.

**Figure 2:**
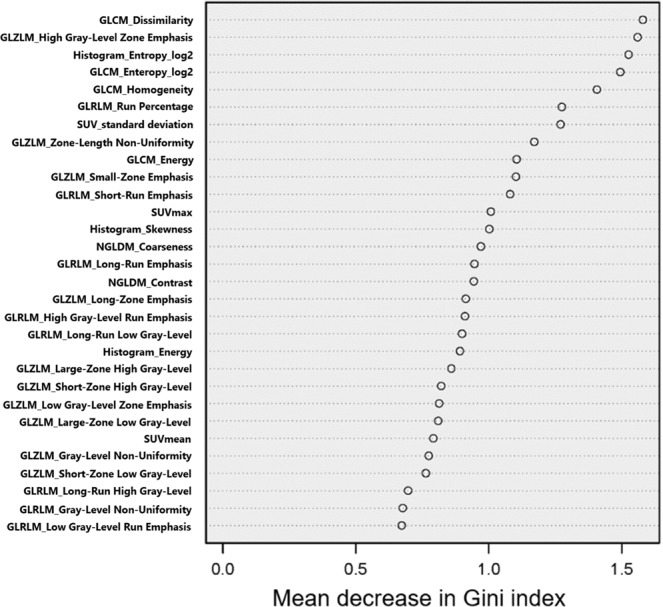
Mean decrease in Gini index showing the importance of PET parameters for the classification between recurrent brain tumor and radiation necrosis in the random forest classifier. GLCM = gray-level co-occurrence matrix; GLRLM = gray-level run length matrix; GLZLM = gray-level zone length matrix; NGLDM = neighborhood gray-level different matrix; SUV = standardized uptake value.

**Figure 3:**
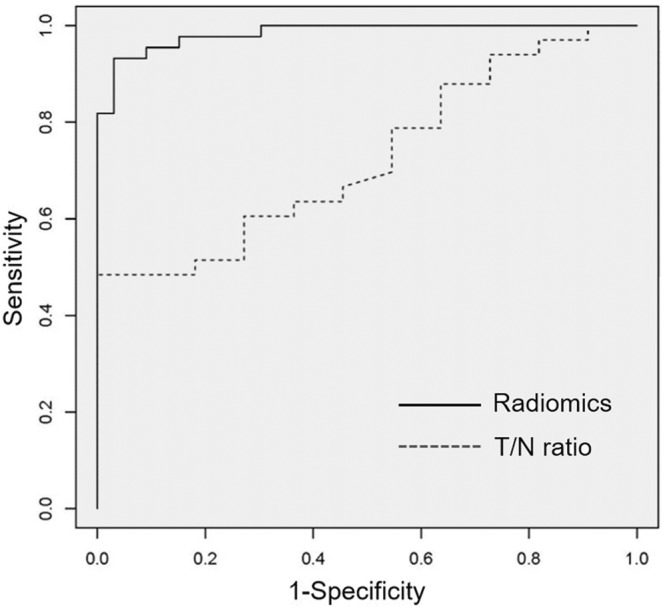
Receiver operating characteristic curve of radiomics with random forest classifier and T/N ratio evaluation for discriminating recurrent brain tumor from radiation necrosis.

Fifty-two lesions detected on MRI were evaluated with MET-PET/CT. Among these, those lesions without definite abnormal uptake (n = 5) or with a volume of interest ≤64 voxels (n = 3) were excluded. Finally, a total of 44 lesions were used for the analysis (Table 1). Among them, 30 (glioma: n = 14, recurrent brain tumor: n = 16) and 14 (glioma: n = 9, recurrent brain tumor: n = 5) lesions were detected on Discovery PET/CT 600 and Biograph 16 PET scanners, respectively. There was no statistical difference for the selection of PET scanner (p = 0.34). Twenty PET parameters showed significant differences between recurrent brain tumor and radiation necrosis. (Table 2). T/N ratio was significantly higher for recurrent brain tumor (p = 0.029) than for radiation necrosis (median T/N ratio [interquartile range]: radiation necrosis, 2.15 [1.85–2.74]; recurrent brain tumor, 2.76 [2.35–3.56]) (Fig. 1a). Among all PET parameters, gray level co-occurrence matrix (GLCM) dissimilarity was the most relevant feature for classification. A box and whisker plot of GLCM dissimilarity comparing recurrent brain tumor and radiation necrosis is described in Fig. 1b. The T/N ratio showed correlation with GLCM dissimilarity with a correlation value of 0.78 (p < 0.001; Fig. 1c). Mean decreases in the Gini index of PET parameters are shown in Fig. 2. The sensitivity, specificity, positive predictive value, negative predictive value and accuracy of radiomics with random forest classifier and T/N ratio evaluation with a cut-off value of 2.83 were 90.1% and 60.6%, 93.9% and 72.7%, 95.2% and 86.9%, 88.6% and 38.1%, and 92.2% and 63.6%, respectively. The area under the receiver-operating-characteristic curves of radiomics and T/N ratio evaluation were 0.98 and 0.73, respectively (Fig. 3).

**Table 1:** Type of lesion with final diagnosis.

Type of lesion	Number of lesions diagnosed as:	
	Recurrent brain tumor	Radiation necrosis
Metastatic brain tumor	15	6
Glioma	18	5

**Table 2:** Comparison of PET parameters showing statistical difference (p-value < 0.05) between recurrent brain tumor and radiation necrosis.

PET parameters*	Recurrent brain tumor (n = 33)	Radiation necrosis (n = 11)	p-value
SUVmax	3.0 [2.7, 3.8]	2.7 [2.1, 2.9]	0.026
SUVmean	1.9 [1.8, 2.1]	1.7 [1.5, 1.7]	0.005
SUV_standard deviation	0.31 [0.22, 0.40]	0.20 [0.13, 0.25]	0.005
Histogram_Energy	0.35 [0.27, 0.44]	0.48 [0.41, 0.59]	0.010
Histogram _Entropy_log2	1.9 [1.5, 2.2]	1.4 [0.92, 1.6]	0.009
GLCM_Contrast	0.93 [0.63, 1.4]	0.45 [0.40, 0.72]	0.006
GLCM_Dissimilarity	0.65 [0.50, 0.77]	0.40 [0.37, 0.55]	0.008
GLCM_Energy	0.15 [0.091, 0.20]	0.26 [0.17, 0.34]	0.016
GLCM_Entropy_log2	3.5 [3.1, 4.2]	2.6 [2.0, 3.2]	0.010
GLCM_Homogeneity	0.71 [0.68, 0.77]	0.80 [0.75, 0.82]	0.010
NGLDM_Contrast (×10^−2^)	3.1 [2.0, 4.7]	1.7 [1.4, 2.8]	0.006
GLRLM_ High Gray-Level Run Emphasis	0.46 [0.38, 0.53]	0.36 [0.28, 0.37]	0.005
GLRLM_ Low Gray-Level Run Emphasis (×10^2^)	2.5 [2.1, 2.9]	3.1 [2.8, 3.7]	0.009
GLRLM_ Long-Run Low Gray-Level Emphasis	0.10 [0.06, 0.15]	0.14 [0.11, 0.24]	0.012
GLRLM_ Run Percentage	0.66 [0.60, 0.72]	0.57 [0.51, 0.65]	0.030
GLRLM_ Short-Run Emphasis	0.73 [0.65, 0.77]	0.63 [0.60, 0.71]	0.010
GLRLM_ Short-Run High Gray-Level Emphasis	31.6 [25.1, 39.1]	21.3 [18.9, 25.3]	0.002
GLRLM_ Short-Run Low Gray-Level Emphasis (×10^−2^)	1.8 [1.5, 2.6]	2.2 [1.8, 2.5]	0.035
GLZLM_ High Gray-Level Zone Emphasis	45.8 [37.8, 70.3]	34.6 [30.0, 38.2]	0.004
GLZLM_ Low Gray-Level Zone Emphasis (×10^2^)	2.6 [1.8, 3.4]	3.1 [2.7, 3.6]	0.032

GLCM = gray-level co-occurrence matrix; GLRLM = gray-level run length matrix; GLZLM = gray-level zone length matrix; NGLDM = neighborhood gray-level different matrix; SUV = standardized uptake value. (*Data represent medians, with interquartile range in parentheses).

## Discussion

The present study appears to be the first to evaluate the diagnostic performance of MET-PET radiomics. MET-PET radiomics showed great diagnostic ability for differentiating between radiation necrosis and recurrent brain tumor, offering superior performance to T/N ratio evaluation. Among the various PET parameters, GLCM dissimilarity was the most relevant feature for diagnosis.

We used a random forest classifier in a machine-learning approach. Random forest is an ensemble approach that computes multiple decision tree-based classifiers using implicit feature selection^21^. In other words, the results offered by a random forest classifier are derived from a combination of various PET parameters, not a single feature such as T/N ratio. Some advantages are seen with a random forest classifier. First, a random forest is fully non-parametric and can therefore be used even if some features show collinearities with other features. Second, overfitting is less of an issue than with other machine-learning methods^21^. These characteristics are particularly beneficial for high-dimensional data analysis such as radiomics using textural features, where not all features are strictly controlled^22^. Indeed, also in FDG-PET radiomics, Ahn *et al*. reported that a random forest classifier provided higher diagnostic performance over other machine-learning algorithms, including a support vector machine and a neural network for predicting the prognosis of lung cancer^20^. The usage of a random forest classifier is likely to have contributed to the excellent diagnostic performance of MET-PET radiomics.

GLCM dissimilarity was the most important textural feature for classification among all MET-PET parameters, including conventional parameters like SUVmax and metabolic tumor volume. GLCM represents how often different combinations of pixel brightness (gray levels) arise in an image, and is classified as a local textural feature^10,23^. GLCM is one of the most robust and reproducible features, and less sensitive to PET scanner variations^24^. GLCM dissimilarity reflects the variation of gray-level voxel pairs, and the value increases if the lesion is heterogeneous^23^. In our study, GLCM dissimilarity was significantly higher for recurrent brain tumor than for radiation necrosis. This indicates recurrent brain tumor showed higher intratumoral heterogeneity than radiation necrosis, and also means heterogeneity was the most important factor for diagnosis, not intensity of uptake. Indeed, other local (e.g., GLCM and neighborhood gray-level different matrix (NGLDM)) and regional (e.g., gray-level run length matrix (GLRLM)) textural features also showed significant differences between recurrent brain tumor and radiation necrosis, representing the higher intratumoral heterogeneity of recurrent brain tumor compared to radiation necrosis. In addition, GLCM showed positive correlation with the T/N ratio, indicating that recurrent brain tumor tends to show a higher heterogeneity accompanying increased activity as compared with that shown by radiation necrosis.

Intratumoral heterogeneity is associated with tumor aggressiveness, treatment response, and prognosis^8,25^. Many studies have demonstrated the clinical value of PET radiomics with textural features for various malignancies^26^. In brain tumor, a small number of texture analysis studies using O-(2-[18 F] fluoroethyl)-L-tyrosine (FET)-PET have been reported^27,28^. FET-PET has been reported to provide comparable diagnostic ability to MET-PET when assessed using conventional PET parameters^29^. Kebir *et al*. investigated the utility of texture analysis on FET-PET with cluster-based analysis for differentiating true progression from pseudoprogression after radiotherapy in high-grade glioma, and found that true progression was related to high heterogeneity^27^. Lohmann *et al*. demonstrated that textural features of FET-PET were helpful for differentiating between radiation necrosis and recurrent brain tumor in patients with metastatic brain tumor, and diagnostic accuracy can be increased in combination with T/N ratio^28^. These results are consistent with our findings. The integration of textural features and conventional PET parameters using the random forest algorithm can boost diagnostic ability. The evaluation with intratumoral heterogeneity by texture analysis is able to complement assessment using conventional PET parameters. In addition, the use of machine learning, as in our study, enables high-level integration of various PET parameters, leading to more robust outcomes^8^.

Several limitations should be considered in this study. First, this was a single-center study and the number of patients was relatively small. This was related to why we did not analyze gliomas and recurrent brain tumors separately. In addition, we selected a cross-validation method to evaluate the diagnostic ability of radiomics classifiers due to small population. At least as of now, the cross-validation method is most commonly used in radiomics studies. However, a training/test scheme, despite needing a large number of examples, is preferable for the validation of classifiers^30^. Second, lesions with a small volume of interest (≤64 voxels) or without significant uptake were excluded from analysis. Although this is inevitable for appropriate texture analysis^31^, these lesions may be considered as radiation necrosis in daily clinical situations. This may be related to the relatively lower diagnostic performance of T/N ratio evaluation with a slightly higher optimal cutoff, as compared to previous studies^4^. Third, two different PET scanners were used. Although these systems were cross-calibrated, differences in reconstruction settings and iteration numbers could have influenced the robustness of textural features^32^. However, this limitation is not specific to our study, and is likely to prove a more challenging issue in large-scale PET radiomics studies across institutions. Recently, some promising harmonization methods, such as ComBat, have been introduced^33^, whose efficacy should be validated in future studies. In conclusion, MET-PET radiomics with a random forest classifier yielded prominent diagnostic ability for differentiating between radiation necrosis and recurrent brain tumor, outperforming assessment with T/N ratio. High intratumoral heterogeneity is likely to be the most important trait for distinguishing recurrent brain tumor. Further study with a larger population using external data is required for translation of the future clinical potential.

## Methods

### Patients

This prospective study was approved by the Institutional Ethics Review Board (National Center for Global Health and Medicine Review Board), and written informed consent was obtained from all patients. Forty-one patients (age, 55.5 ± 13.2 years; 21 males, 20 females) with brain tumor (glioma, n = 20; metastatic brain tumor, n = 21) who had been treated with radiation therapy (either conventional radiotherapy or stereotactic radiosurgery) and had undergone MET-PET/CT between October 2014 and June 2016 were included in this study. Before MET-PET/CT, all patients had undergone brain MRI depicting one or more tumor or tumor-like lesions. The primary malignancies for metastatic brain tumors were lung (n = 13), breast (n = 4), head and neck (n = 2), and colorectal (n = 1) cancers, and pheochromocytoma (n = 1). Patients with glioma included World Health Organization histopathologic classifications of grade 2 (n = 4), grade 3 (n = 8), and grade 4 (n = 8). Median interval between radiation therapy and MET-PET/CT was 13.5 months (interquartile range: 7.9–29.2 months). The gold standard for diagnosing radiation necrosis or recurrent brain tumor was: (1) pathological diagnosis using specimens obtained by biopsy or surgical resection; or (2) more than 6 months of follow-up with MRI. Lesions were defined as radiation necrosis when spontaneous shrinkage was identified, and as recurrent brain tumor when stable or showed an increase in size.

### MET-PET/CT examination

Each patient was injected with MET (384.0 ± 22.7 MBq) 20 min before initiating a 10-min emission scan. Patients underwent PET/CT using either of two cross-calibrated PET/CT systems (14 patients: Biograph 16; Siemens Medical Solutions, Erlangen, Germany or 27 patients: Discovery PET/CT 600; GE Healthcare, Pewaukee, WI). Patients were randomly allocated to each scanner. CT data for the Biograph 16 and Discovery PET/CT 600 were acquired at 120 kVp using an auto-exposure-control system, beam pitches of 0.80 and 0.94 and slice thicknesses of 5.0 mm and 3.8 mm, respectively. CT images were used for attenuation correction as well as image fusion. PET images were acquired in three-dimensional mode, and reconstructed with an ordered subset expectation maximization algorithm: 3 iterations and 8 subsets for Biograph 16; and 3 iterations and 16 subsets for Discovery PET/CT 600. A Gaussian filter of 5-mm full-width at half-maximum was used as a post-smoothing filter.

### Image analysis

**Figure 4:**
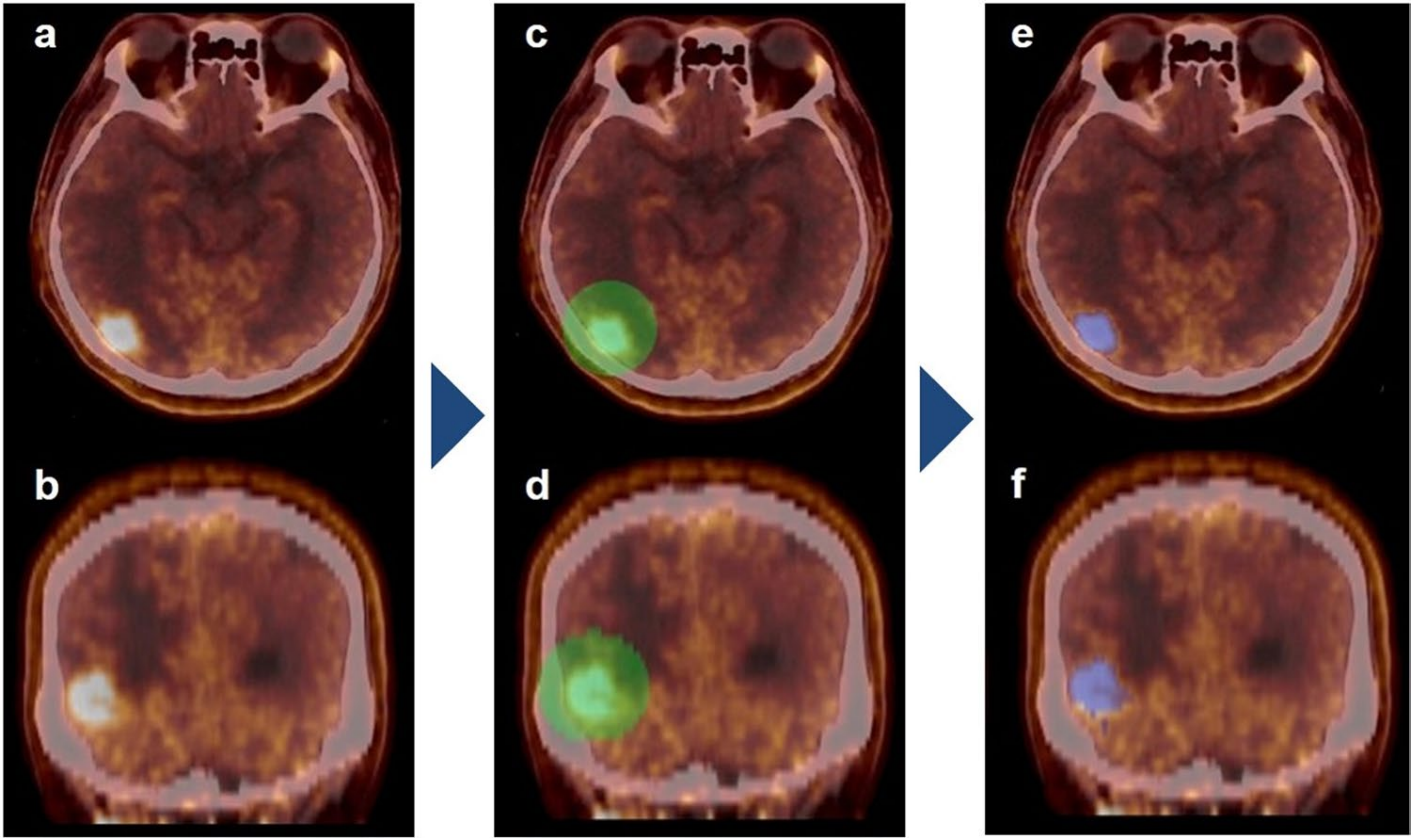
A 59-year-old man after stereotactic radiation therapy to a brain metastasis from lung cancer. Axial (**a**) and coronal (**b**) MET-PET/CT show focal uptake on the right occipital lobe. (**c**,**d**) A 3-dimensional sphere is set to encompass the lesion. (**e**,**f**) A volume of interest with a threshold of SUV of more than 1.3-times that of the contralateral normal frontal brain cortex is obtained and used for texture analysis.

Images from MET-PET/CT were evaluated by two experienced nuclear medicine physicians (M.H and R.M) with reference to MRI. The physicians were blinded to the final diagnosis of the lesions. First, SUVmean of normal brain cortex in the frontal lobe (contralateral to the lesion) using a 1.0-cm^3^ sphere was measured. After reaching consensus of the target lesion by the two nuclear physicians, the three-dimensional volume of interest was then set to encompass the lesion, and the area showing an SUV >1.3-times greater than normal brain cortex (SUVmean) was automatically contoured (Fig. 4). All image analysis was performed using the LIFEx package^34^. The threshold for setting the volume of interest was used after referring to previous studies^35,36^. We applied absolute resampling using 64 gray levels (size bin = 0.317) between 0 and 20 SUV units using the LIFEx software. Lesions showing a volume of interest ≤64 voxels or no definite abnormal uptake were excluded, because textural features cannot be accurately quantified for small lesions^31^. A total of 42 PET parameters including conventional (e.g., SUVmax and metabolic tumor volume) and textural features were extracted. T/N ratio was measured by dividing the SUVmax of the lesion by the SUVmean of the contralateral normal cortex.

### Machine learning and statistical analysis

A random forest classifier was trained to separate radiation necrosis from recurrent brain tumor after balancing the number of lesions in each state by using the synthetic minority oversampling technique with the “DMwR” R package^37^. Features were centered and scaled prior to data augmentation using the synthetic minority oversampling technique, which increased the number of minority class to the majority class^38^. Random forest analysis was performed using the “randomForest” R package. The RF classifier was optimized for the number of trees (ntree) (100, 250, 500, 750, 1000, 1500) with repeated (n = 100) and 10-fold cross-validation using the “caret” R package, and optimal ntree and number of variables at each split (mtry) were determined (ntree = 100, mtry = 1). The diagnostic performance of the RF classifier was assessed using a 10-fold cross-validation: Patients’ data were randomly separated into 10 cohorts, with 9 of them used for training, whereas the remaining cohort was used for testing. All tests were run 10 times with the average value reported as the performance. To assess which PET parameters are important for classification, mean decrease in Gini index was evaluated with the trained random forest model. Gini index is an efficient approximation of entropy in a computational manner. It is calculated at each node split of the RF and reflects how well the data could be split into two classes at a particular node in each tree. Gini index measures the degree or probability of a particular variable being wrongly classified for each feature at a node^21^. The diagnostic performances of radiomics and T/N ratio evaluations were assessed using sensitivity, specificity, positive predictive value, negative predictive value, and accuracy as well as area under the receiver-operating-characteristic curve. The optimal cut-off value for T/N ratio was determined by receiver-operating-characteristic curve analysis. For comparisons of PET parameters and T/N ratios between recurrent brain tumor and radiation necrosis, the Mann-Whitney U test was used. The correlation between the T/N ratio and the most relevant PET parameter was assessed using Spearman’s rank correlation coefficient. The level of significance was defined as a p-value of less than 0.05.

### Ethical statement

This study was approved by the local Ethics Committee and was carried out in accordance with the 1964 Declaration of Helsinki. Written informed consents were obtained from all subjects for participation and publication of this report.

## Data Availability

The datasets analysed during the current study are available from the corresponding author on reasonable request.
